# Expanding Research Integrity: A Cultural-Practice Perspective

**DOI:** 10.1007/s11948-021-00291-z

**Published:** 2021-02-09

**Authors:** Govert Valkenburg, Guus Dix, Joeri Tijdink, Sarah de Rijcke

**Affiliations:** 1 Centre for Science and Technology Studies (CWTS), Faculty of Social and Behavioral Sciences, Leiden University, Leiden, Netherlands; 2 Department of Ethics, Law and Humanities, Amsterdam University Medical Center, Location VUmc, Amsterdam, Netherlands; 3 Department of Philosophy, VU University, Amsterdam, Netherlands; 4 Present Address: Department of Interdisciplinary Studies of Culture, Faculty of Humanities, Norwegian University of Science and Technology, Trondheim, Norway; 5 Center for Higher Education Policy Studies (CHEPS), Faculty of Behavioural, Management and Social Sciences (BMS), University of Twente, P.O. Box 217, 7500 AE Enschede, the Netherlands

**Keywords:** Research practice, Research culture, Institutions, Research integrity, Research ethics

## Abstract

*Research integrity* (RI) is usually discussed in terms of responsibilities that individual researchers bear towards the scientific work they conduct, as well as responsibilities that institutions have to enable those individual researchers to do so. In addition to these two bearers of responsibility, a third category often surfaces, which is variably referred to as *culture* and *practice*. These notions merit further development beyond a residual category that is to contain everything that is not covered by attributions to individuals and institutions. This paper discusses how thinking in RI can take benefit from more specific ideas on practice and culture. We start by articulating elements of practice and culture, and explore how values central to RI are related to these elements. These insights help identify additional points of intervention for fostering responsible conduct. This helps to build “cultures and practices of research integrity”, as it makes clear that specific times and places are connected to specific practices and cultures and should have a place in the debate on Research Integrity. With this conceptual framework, practitioners as well as theorists can avoid using the notions as residual categories that de facto amount to vague, additional burdens of responsibility for the individual.

## Introduction

*Research Integrity* (RI)^1^ as an umbrella concept captures a collection of qualities that researchers and research institutions must possess, to ensure that research produces valid and reliable scientific knowledge, in a way that is societally desirable, and with a proper positioning of scientists in society. The concept designates two primary subjects that are “to do” integrity: the researcher and the research institution. We argue that alongside these two subjects, a third one merits further attention: the *culture* or *practice* in which researchers do their work. Giving further substance to these concepts enables actors to target interventions that can help build RI more specifically.

The current literature predominantly addresses institutions and individuals as relevant subjects in integrity work that can be accountable and responsible for promoting RI. For example, most advice on RI, such as the 11 recommendations in the report by the Committee on Assessing Integrity in Research Environments (2002), consists of standards of good research behaviour that the researcher should live up to, or of structural measures that institutions have to provide. In addition, a review of a decade of empirical research on research integrity revealed that empirical analysis is skewed towards measures that target individual researchers, and pays less attention to the effect of institutional governance and policy (Aubert Bonn and Pinxten 2019). What is more, the authors remind us that individual researchers are likely to act differently in specific situations, as their perceptions and expectations will be different. This diversity has so far been poorly addressed in existing research.

On the one hand, values can be thought of as essentially individual duties (Meriste et al. 2016; Steneck 2006; Shamoo and Resnik 2015), as they often refer to “doing good.” Thereby, they almost tautologically appear as a trait that should be internal to the researcher as a person. Also, surfacing mishaps are usually judged as a failure of individuals to comply with obvious norms of integrity—the proverbial “rotten apples” that spoil the bunch. In addition, the individualization of such responsibilities is reflected by the fact that courses on RI are typically offered to individuals. We never send an academic hospital to ethics class—in a manner of speaking. And finally, the responsibility for realizing more specific values constituting RI are often attributed to individual researchers by research codes of conduct, even though values such as transparency, respect, and responsibility could equally as well be seen as part of the responsibility of a decent institution, and such attributions are indeed often ambiguous (Valkenburg et al. 2020). Only until recently, the revised Dutch Code of Conduct addresses the responsibility of an institution and refer to it as institutional duties of care (p. 20).

On the other hand, considerable attention is directed at arranging organizational structures in such a way that integrity is safeguarded independently of the actions of individual persons (DeMets et al. 2017; Israel and Drenth 2015; Committee on Assessing Integrity in Research Environments 2002; Kaiser 2014; Jordan 2013). Research institutions are expected to put in place rules and regulations regarding integrity and responsible conduct. They are typically supposed to have committees and boards that assess allegations of misconduct. Universities and medical schools offer ethical training for staff at all levels. And institutions as well as professional societies issue Research Integrity codes of conduct that their employees or members are supposed to follow. RI has thus become an object of governance: something that institutions are called to account for, and that they seek to marshal their employees into.

Reality is obviously much more complex than a simple divide between individual and institutional responsibilities for the realization of values, even though for example the Committee on Assessing Integrity in Research Environments (2002) does phrase advice along these sharp lines.^2^ Arguably, individuals and institutions constitute a complex adaptive system (National Academies of Sciences Engineering and Medicine 2017, p. 14), and the boundary between the two categories is not that clear-cut.

The interrelations between actors and their contexts, i.e. how actors respond differently to the signals provided by their environment, has been argued to be a less central topic in the literature (Aubert Bonn and Pinxten 2019). Nonetheless, there are several studies that do focus on the ways in which individuals are situated in their respective institutional contexts. To start with, there are studies that focus on perceptions of the organization and on environmental stressors, where these “perceptions” are in some literatures reckoned the constituents of “research climate”. Similarly, individuals are less able to resist illegitimate coping strategies when they cannot realize personal ideals or receive persistent negative feedback (Martinson et al. 2010, 2006).

In addition, there are studies that identify mentoring as an important site for novices to learn and incorporate the norms of the professional community. Anderson et al. (2007a) show that problematic behaviour can be related to mentoring on research ethics, securing of funding, and academic survival. It is interesting that they observe that mentoring on different topics leads to different levels of RI promotion. Notably, early-career researchers seem more (rather than less!) likely to engage in problematic behaviour when they are mentored on financial issues seems to *increase* the likelihood of problematic behaviour in early-career researchers.

Finally, there are studies that direct attention at patterns of action. For example, the National Academies of Sciences Engineering and Medicine (2017) list “a larger pattern of social deviance” as a source of problematic behaviour that may compromise individuals’ research integrity. The report draws on Reason (2000) in arguing that the most reliable organizations—think of nuclear power plants and air traffic control rooms—are those that build structural safety measures that circumvent such human fallibility. In contrast to how such organizations are organized, the marketization and commercialization of current research institutions leads to competition that compromises researchers’ integrity by prioritizing their own interests above those of the scientific community (Anderson et al. 2007c, 2007b).

This body of work has in common the suggestion that RI is linked to less tangible things than individual responsibilities and organizational rules and regulations. Carrying this idea further, we make an additional step of conceptualizing the relation between the individual and the collective. We do so, first, by thinking through “culture” and “practice” as twin notions that mediate the relation between individuals and the institutional contexts in which they act. These two notions merit further scrutiny, in particular how they are different from both “the institution” and “the individual”. Using insights from social practice theory and cultural theory, we hold that advanced notions of culture and practice should be central to RI debates and interventions.

In the section thereafter, we link our notions of culture and practice to the norms and values that typically appear in discussions on RI. We divide them in two broader categories. On the one hand, there are sanctionable norms and values that lead to individual and organizational responses when researchers do not live up to them. These values include the avoidance of falsification, fabrication and plagiarism (FFP), fair credit, transparency and human dignity. On the other hand, there are aspirational values that are less easily sanctioned by others. These values have to do with things that are good to do and that make one a good or better scientist if one holds them dear, but where there is no way to formally enforce them. We then show that both categories of values take us beyond the individual and institutional levels of analysis and intervention. In fact, we suggest that a vast proportion of what makes up research integrity is negotiated and constructed at these levels of culture and practice.

In the final section, we conclude with recommendations for how RI measures can benefit from further developed notions of practice and culture and how implementing RI can be targeted better at specific sites of intervention.

## Thinking through Culture and Practice

### Culture, Practice, and Climate in RI Literature

With our claim that culture and practice need further development we do not mean to argue that these notions are not discussed at all. In fact, references to notions such as “culture”, “practice” and “research climate” abound. For example: the term “practice” is used to place a practice-based ethics in opposition to a principle-based ethics (Nia et al. 2019; Fuerholzer et al. 2019; Clegg et al. 2007). “Culture” emerges in notions such as a “peer review culture” (Atkinson 2001), a “culture of publish-or-perish” (Genova and de la Vara 2019), or “national culture” (Antes et al. 2018). The contribution by Meyers (2004) effectively equates culture with what we have earlier defined as the institutional level, i.e. the norms and standards set by leadership and enshrined in rules and regulations. Also, in explicit relation to research integrity, it is broadly recognized that “culture” is crucially important to promoting research integrity (e.g. Bouter 2015; Martinson et al. 2005; DeMets et al. 2017). Ann Nichols-Casebolt (2012, p. 16) substantiates a “culture of integrity” as making sure ideas of integrity are part and parcel of education, discussions, having clear mission statements, setting specific requirements, setting policies for reporting misconduct, and setting good examples. Ellis (2015) identifies research culture as the realm where perverse publication incentives compromise integrity, notably through specific reward structures. And finally, in Anderson et al. (2007a), the notion of culture emerges chiefly as a normative ideal of science, that is handed over to the individual through mentorship and education.

While these accounts of culture can roughly be understood as notions of “what culture does”, accounts have also been given of “what culture consists of”. For example, the report by the National Academies of Sciences Engineering and Medicine (2017) presents a number of elements through which culture can be operationalized for research. These include a range of what could be called “good behaviours,” including proper data handling, publication, correcting errors, collaboration, and peer review. The report also mentions incentives that run counter responsible research, such as publication pressure and the need to acquire funding, which arguably make up a *bad* culture.

The report by the Committee on Assessing Integrity in Research Environments (2002) defines (p. 60) culture as shared norms, values, beliefs, and assumptions, and climate as the prevailing moral beliefs. Yet, its operationalization (p. 54) is limited to what we prefer to regard as institutional: clear definitions of roles and responsibilities, proper policies and procedures, and thoughtful decision-making practices. To the question how this is to be effectuated in practice and in the actual actions of researchers, only “leadership”, “supervision” and “socialization” are mentioned. This calls for further development beyond giving merely conceptual advice.

In some RI literatures, climate is explicitly distinguished from culture. Research climate has been operationalized as individual and shared perceptions of the research culture (Crain et al. 2013; Martinson et al. 2016). This definition singles out climate as the more tangible and observable correlate of culture. In this conceptualization, climate is split into categories such as visible ethical leadership, openness to ethical discussion, conformity to policies, and the awareness that ethical behaviour is expected. Martinson et al. (2016) mention that this conceptualization of climate is more subjective in the sense that it engages with the *perception* that individuals have of the research culture. This at least potentially opens up the hazard of implicitly rendering all responsibility to the individual level: after all, it is the individual who has to act on these impressions. This calls for complementary thought of how such cultures operate more independently of how people perceive them. Even if we assume that cultures can only operate to the extent of what people make from them, it is not necessarily the case that these people have an explicit or even coherent account of how they perceive them, nor is their perception necessarily in congruence with how it actually works out. For example, people may think of their work sphere as very much conducive to plagiarism and corner cutting, while in fact neither they themselves nor their colleagues actually commit this transgression.

In our view, these notions of culture (and climate) and practice leave some of the potential of these concepts unrealised. They merit further development, and the question should be asked explicitly what culture and practice can (help to) explain that cannot be explained at the level of individuals performing well or poorly, nor at the level of institutions being arranged properly or improperly.

### Culture

Clifford Geertz possibly offers the most foundational and widespread notion of culture. He defines culture as “an historically transmitted pattern of meanings embodied in symbols, a system of inherited conceptions expressed in symbolic forms by means of which men [sic] communicate, perpetuate, and develop their knowledge about and attitudes toward life” (Geertz 1973, p. 89). Swidler (1986) adds that culture also appears as *ritual* in the literature and in general conversations: the recurrent social processes through which behaviour is shared. Following Keesing (1974) and Hannerz (1969), Swidler (1986) adds that there is something public and explicit about how meaning circulates as the constituent of culture. The primary importance of culture to RI is in that it is what orients people’s actions (Eckstein 1997).

Relating more specifically to research settings, Knorr Cetina (1999, p. 10) has argued that cultures, at the level of research practices, engender specific styles of knowledge production, and therefore need to be attended to when explaining the production of scientific knowledge. She posits that three properties can be attributed to such research cultures. The first is that they are *not uniform* but may differ across practices and disciplines. Second, culture comes with a certain *richesse of what matters to courses of affairs*, including instrumental, linguistic, theoretical and organizational frameworks. And third, it relates to the patterns of meaning through which people communicate, which are hand over to next generations (cf. Geertz 1973). This is why, according to Sismondo (2008), research outcomes are heavily marked by the research context in which they come about.

To operationalize culture further as an orientation of people’s actions with respect to RI, it seems meaningful to split this orientation tentatively in four parts. First, it may be thought to predispose people to *do* particular things: following routines and habits, copying behaviour et cetera. Second, it may predispose people to *value* things in a particular way: what is important, what is right or wrong, and what is it that a good researcher typically does. Third, it may predispose how people *know* things, including but not limited to the disciplinary curricula that we consider part of the theoretical frameworks mentioned by Knorr Cetina. And fourth, it may pre-structure distributions of responsibility and accountability: who does a given task belong to, who or what can we expect to solve a problem, and who can we summon in case things go wrong. We will use these dimensions of culture and practice to assess a number of values below.

### Practice

The notion of practice directs attention at an empirically existing situation in which people operate, in this case the practice of research. As follows from the foundational text by Pickering (1992), studying scientific research practice makes properties of science into *explanandum*, rather than seeing for example different disciplines as *explanans* of scientific outcomes. That is to say: we cannot use scientific knowledge or its nature as the explanation of why science happens to be done the way it is, but rather we must look at how science is actually done, if we want to understand the nature of scientific knowledge. In the context of RI, such a reversal would lead us to asking not so much what a good scientific conduct is and derive the answer from ethical and other normative principles, but rather to asking how such standards have been put in place, and the negotiations needed to both define and enact such ideas of integrity.

In its most basic form, a practice is any unit of coordinated human action. Reckwitz (2002, p. 249) defines the basis of practice as a “routinized type of behaviour which consists of several elements, interconnected to one other: forms of bodily activities, forms of mental activities, “things” and their use, a background knowledge in the form of understanding, know-how, states of emotion and motivational knowledge.” MacIntyre (1981) considers it vital that there is some shared understanding of a good that the practice pursues (see also Schatzki 1996, p. 89), but not all notions of practice are that strict in the necessity of aspiring to a shared good for a practice to exist. In the case of scientific research practices, the production of valuable knowledge could be surmised to be such a shared good, but we do not take this to be an essential or defining property for the current argument.

Social practice theory has been positioned primarily as an alternative level of analysis to more structuralist social theories, and builds on influential authors such as Bourdieu, Giddens, Taylor and the later Foucault (see Reckwitz 2002). Schatzki (1996) already elaborated that a level of analysis between the individual and any sort of “social totality” had so far been lacking in social theory. In between these two levels, various versions of practice theory offer an alternative level of explanation of what determines human action, as opposed to explanation from either mere principles or mere goal orientation (Reckwitz 2002).

Despite their diversity, notions of practice do share a number of elements. One is that practices are situated in *space and time*. The place aspect is that practices are connected to specific sites, and spatial proximity is vital for people to become performers of the practice. The time aspect refers to their repetitiveness and path-dependency in the sense that what has happened before is of constituting significance to what happens now. According to Pickering (1992), this temporal aspect is in fact where practice is complementary to culture. Only by focusing on specific times and places can meaningful observation take place, and becomes apparent how peoples’ actions are (also) driven by routines, workplace facilities, colleagues, etc. (Schatzki 1996, p. 89). Practices differ across time and place- also within overarching institutions. This resonates with the work by Knorr Cetina (1999), pointing towards the differences that exist between how different scientific disciplines produce knowledge in different ways.

Thinking of human action as happening in practices and to a smaller or larger extent determined by those practices, also means a move away from seeing actions as purely individual phenomena. This is not a mere reduction of human action to “structure” or any other concept located outside the individual. The level of practice connects those actions to the context in which the individual is situated (Schatzki 1996, p. 97), and members of a practice also take part in the production of those practices through their performance (Shove 2014; Douglas 1986, p. 45). The constituting relationship between practice and human action is thus bidirectional.

Apart from time and space, a second element that different notions of practice share, is the *articulation of how technologies and other material arrangements affect* people’s actions. The working of devices cannot be seen apart from the actions of human beings, and this is where *skills* come in as an essential element of practices: what people are capable of, both mentally and physically. This is also pivotal in setting the boundaries of the practice: mastering specific skills to engage with relevant devices becomes a condition for being admitted as a member of the practice (Shove 2014, 2017). Skills are importantly connected to the repetitiveness of a practice. Many skills are transferred from masters to apprentices, and often implicitly so, by performing them time and again. Practices are thus among the primary sites where mentor-apprentice relationships emerge.

This point generalizes to the idea that practices have a scheme of *membership*: not just everybody takes part in any given practice, and it requires a degree of socialization to become accepted as “one of them”. Defining who is “in” and “out” is vital for the practice to survive, and the accompanying process of socialization is an important mechanism through which the practice and its culture predispose members to do, value, know and account in specific ways.

## Research-Integrity Values in Practice

Continuing our argument of splitting the workings of culture and practice into four dispositions of doing, valuing, knowing and accounting, we propose to distinguish between two main categories of norms and values. On the one hand, we discern those norms and values that are sanctionable. One typically experiences unfavourable consequences if one does not live up to them. On the other hand, we discern those norms and values that are aspirational: things that are good to do and that possibly make you into a bad scientist if you don’t hold them dear, but where there is no formal way to enforce them. The rationale behind this tentative classification is that sanctionability naturally places an issue at the institutional level: it is literally enshrined in rules, regulations, procedures and formal responsibilities of offices how sanctions are shaped and executed. Thus, if the world only consisted of institutional and individual responsibilities, sanctionability would be an informed guess of where the boundary is. This rationale thus guides our inquiry into culture: as a working hypothesis, sanctionable values are a concern of institutions and management, whereas aspirational values are a concern of research scientists and the research communities they work in.

One important proxy question to this boundary condition is who or what suffers in case the value is breached, which provides a direct answer to the question of accountability. With aspirational values, the consequences of breaching are primarily for the researchers; they will typically suffer a loss in reputation. Conversely, when breached, sanctionable values lead to liability for the institution, damage for eventual patients or research subjects, or a corruption of the body of scientific knowledge (see Shaw 2019 for a treatment of this last point). Thus, the question whether a value is aspirational or sanctionable also depends on the distribution of benefits, ownership and liability, and hence power, between the researcher and the institution.

The distinction between sanctionable and non-sanctionable values is compatible with the observation by Horbach and Halffman (2017), who show that sanctionable values are more the language of policy makers and journalists, whereas aspirational values appear more in the language used by scientists themselves. Similarly, Israel and Drenth (2015) note that aspirational values fall behind in terms of their effectuation in practice. Finally, it resonates with the observation by Davies (2019) of a tension between ideals of good science that researchers aspire to, and the abstract, principle-based codes that seem not to capture these ideals. The exact distinction between sanctionable and aspirational values remains contingent, and consequential for what practice will prevail, which is exactly why this level of practice merits further explanation in RI theory.

### Sanctionable Values

Sanctionable values are in a way the “hard boundaries” of what gets defined as proper scientific research. According to Plemmons et al. (2006), knowledge of these principles is successfully conveyed in RI courses. One could think of the avoidance of fabrication, falsification and plagiarism. Other clear examples are the proper use of informed consent in case of medical research, and the principle in the engineering sciences not to accept assignments for which one lacks the proper qualifications. Also, we may somehow expect these hard boundaries to play out in explicit ways in who is included in the practice or not.

In the following, we highlight four values that circulate primarily as sanctionable. The list is not exhaustive and even to some extent arbitrary. The items are merely intended to exemplify how such sanctionable values can be thought to connect to substantiated notions of practice and culture.

#### Avoidance of Falsification, Fabrication and Plagiarism

Falsification, fabrication and plagiarism (FFP) count as the epitomes of a lack of RI. Through plagiarism, credit is withheld from the people who have actually done the research. And through fabrication and falsification statements enter the scientific knowledge base that are in fact untrue (Shamoo and Resnik 2015, p. 38). Such cases are typically resolved through institutional measures, but it is worth asking how FFP can emerge, in light of the above definitions of practice and culture. Perhaps there are circumstances that at least enable people to “give it a try” to get away with improper behaviour—even though today, most institutions and publishers have access to some form of plagiarism check (Luparenko 2014). Though these automated checks are not (and probably will not very soon be) perfect, it requires skills and intricate knowledge of the whole chain of scientific knowledge production to get away with plagiarism. These chains of knowledge production are discipline-specific and practice-specific. Hence, in order to stand a reasonable chance at successful plagiarism, *one has to be a member of the practice* in the first place.

A similar argument can be made about falsification and fabrication. If researchers want made-up knowledge to appear credibly, they need intricate knowledge of how their claims will be assessed in the peer-review process. This knowledge is only available in the practice itself, and can only be learnt in the same way other skills are transferred in practice: through mentoring, practising, and various forms of teaching.

This means that apart from the obvious sanctioning of FFP-related misconduct, the ways in which the practice itself makes such conduct possible in the first place, could be subject to further reflection. In a way, the usual training is a perfect preparation to actually *commit* the transgression. Carrying this to a conclusion on a substantiated notion of practice, it could be suggested that the master-apprentice relationships in which the skills are transferred, could do with more reflection on how such skills can (and should not) be abused. Similarly, the repetitiveness of practices could be taken as an object of reflection in case misconduct emerges: what where the patterns of action that led to the misconduct, or at least have failed to eliminate it? Has anything been lacking in those patterns that could over time have served as an additional safeguard against the mishap taking place? And, to relate to the different roles that “things” can have in a practice: is there any way in which the infrastructure of automated plagiarism checks could have been used or arranged differently, so as to improve its performance (possibly combined with additional human skills), to prevent plagiarism?

In terms of the questions of culture and practices, it is clear that even if a practice does not force a person to “do” FFP, it at least enables them to. At the same time, material entities like plagiarism checks counter this ability to some extent. Also, the practice expresses an ambiguous valuation of cutting corners: it should not be done, but if successful, it may help one’s career move on.

#### Fair Credit

Closely related to the problem of plagiarism is the fact that scientists are assumed to be fair about what is their own merit, and what is the work of others. Authorship should be attributed to the people who have actually deserved it through their work. People should also be credited through other means, for instance by citing their work (Plemmons et al. 2006). Consoli (2006) shows however that the category of “author” is far from unproblematic: notions such as “responsibility for the output” and “relative contribution to the output” are hard to quantify or compare to some sort of threshold. Also, it is clear that many aspects of the incentives and rewards for authorship that define the landscape in which publishing takes place (Martinson 2017), are in fact such that fair credit is in fact not always an attractive way to go. In addition, there are very clear power relations between seniors and juniors that disturb practicing fair credit (Shah et al. 2018). Thus, the meaning of the category “author” is not self-evident and univocal, which means that it will receive different specifications in different contexts. Taking and giving credit corresponds directly to the distribution of accountability and responsibility. This is thus in fact a mechanism through which culture may play a more important role than institutional relations or individual virtue, and socialization into a practice reproduces it.

Thus, the intricacies of fair credit and the diversity of practical implementations of it, clearly form a clue towards where a culture may prioritize a specific valuation over others. Also, who is accountable for the exact acknowledgement of credit will differ between practical situations: in some disciplines, hierarchy is such that research leaders are co-author by default, and others are not. At exactly this point, Thornton (2013) argues that entitlements are dominantly shaped by masculine and neoliberal norms.

#### Transparency

Research should be transparent, or so the consensus can be assumed to be. An editorial in Nature (2017) provides 5 steps to substantiate transparency: pre-registration or publication of a research protocol prior to conducting the research; pre-publishing a draft before final submission of the paper; releasing the data analysis plan; releasing the analysis code; and publishing the data set. It needs saying that these steps are deeply ingrained with a biomedical and natural science approach, and generalizing them to other fields, notably social sciences and humanities, might involve some critical and problematic translation. What such steps would look like in a strictly theoretical exercise like mathematics, or for example in anthropology where anonymity and confidentiality are key to the production of data in the first place, remains to be debated (see also Penders et al. 2019; Irwin 2018). For example, Spier (2006, p. 189) emphasizes the need for rigor in method and reporting. It is at this point already instructive, as Consoli (2006) argues in reference to the US *Federal Policy on Research Misconduct*, that the presentation and publication of proper facts is- in that policy—considered a more important responsibility than the exact conduct in the lab that precedes that very publication. Remarkably, in a large-scale study on how scientists conceive of good research practice, Hangel and Schickore (2017) argue that especially the reporting of method often remains notoriously obscure. They also show that transparency of primary material is often obfuscated, for example by working with numerical codes that nobody can decipher.

Regarding transparency, the answers to the questions of action, value, knowledge and accountability are ambivalent. It makes an individual accountable, but forces to give up any competitive edge related to knowledge ownership, which is a particular way of valuing. Also, the elegant presentation of e.g. methodology is a skill that requires training, which likely comes with mentorship and jargon, and the membership that is constructed through those. Transparency is thus ambiguous, and it is crucial here that this ambiguity cannot be resolved by clearer (institutional) rules, nor by (individual) moral deliberation, which thus makes the accountability in fact ambiguous. Thus, even if transparency appears sanctionable, it depends on the practice context how it unfolds exactly.

#### Human Dignity

Perhaps the most ambiguous value in the category of sanctionable values is that of human dignity. On the one hand, it emerges as strongly sanctionable, from historical failures such as the Tuskegee experiment (Brandt 1978; Daugherty-Brownrigg 2013) and the atrocities of research in Nazi concentration camps (Baumschlag 2005). At the same time, standing definitions do not help us very much. For example, Drenth (2006, p. 17) defines dignity as the safeguarding of all individuals’ autonomy and freedom of choice, which in the case of participation to research is chiefly shaped as informed consent, and the rejection of every intent to commercialise the human body. Similarly, Spier (2006, p. 191) defines it as the avoidance of any intended negative effects on the environment and society, both for current and future generations. In a general sense, dignity has been observed to be a term that is utterly vague, and usually captured to defend very particular interests (Macklin 2003; Pinker 2008).

Hence, in addition to the aforementioned procedural implementations, it seems that dignity importantly remains a matter of “good intuition”. While this may be more open to individual moral insight, compared to for example transparency, it is also a matter of how the Tuskegee and Nazi stories circulate in courses and mentorship relations. Thus, this is a matter of how people “know” things, including knowing in a particular way how their research relates to the obvious atrocities. Also, the translation of these stories to concrete decisions on the work floor is dependent on the “doing” and “valuing” at specific times and places, in ways that cannot be reduced to institutional rules nor individual qualities.

#### From Sanction to Practice

Even though we started the present set of examples as a tentative list of sanctionable values, in all cases there are sides to them that are not resolved by sanctioning or other institutional arrangements. The realization of these values depends on how routines circulate, how actions are valued, how responsibilities are distributed between people, and between people and the institution. It also, in some cases, depends on the practice-based skills with respect to research devices as well as (working around) plagiarism checks. In all the values listed here, we see that the responsibility for their realization is not reducible to either the individual person, or the institution.

### Aspirational Values

Starting from the assumed sanctionability of the values above, we observed that there are in fact more cultural and practice-related aspects to them then might be suggested by their initial appearance as sanctionable and the according institutional responsibility to secure them. What does it look like if we start from the other end of the spectrum, i.e. values that appear as aspirational and hence connected to individual responsibility? One could think of honesty, scrupulousness, independence and responsibility (KNAW 2018). These are said to be less successfully conveyed in RI courses (Plemmons et al. 2006). Shamoo and Resnik (2015, p. 283) argue that beyond avoiding harm, scientific research should be aimed at furthering the public good and public knowledge. They conclude that little substantiation has been given thereto so far, which we take to be a hint at their substantiation taking place in practice. Following this line of thought, we discuss four such aspirational values and how this substantiation can be understood.

#### Integrity

It may appear circular to discuss “integrity” as a constituting value if it is also the overarching goal. Clearly, the sanctionable values above are part of it. Nonetheless, notions of integrity proper do circulate in much the same way as aspirational values do. For example, Becker (1998, p. 157) as quoted in Breit and Forsberg (2016, p. 15), understands integrity as “the principle of being principled, practicing what one preaches regardless of emotional or social pressure, and not allowing any irrational consideration to overwhelm one’s rational convictions”. A lack of integrity (ibid.) consists of lack of principles; lack of consistency in moral principles; and behaviour influenced by social pressures. In other words, integrity is the capacity to act in accordance with moral principles, but those moral principles themselves are not further substantiated, or at least not within this definition.

The substantiation that such openness calls for is by no means essentially the responsibility of the individual, the institution, nor essentially the product of culture and practice. Rather, it will be a combination of those, and the balance may be tipped differently in different cases. Nevertheless, discussing the value of integrity here is instructive: it offers a clear example where limiting the analysis to individuals and institutions would overlook the importance of how knowing, doing and valuing are predisposed in practice.

#### Inquisitiveness

Many sources mention inquisitiveness and curiosity as primary virtues for scientists (Shamoo and Resnik 2015; Drenth 2006; Gläser et al. 2002). At face value, this appears as an predominantly personal trait. Yet, Shamoo and Resnik (2015, p. 61) argue that choice of research topics, so what *exactly* the scientist practices curiosity on, is inextricably tied up with the resources that are available for doing research. This renders them ambiguous as a personal responsibility: it is equally the institution’s responsibility to provide resources. Thus, institutional responsibilities clearly extend beyond the prevention of problematic behaviour. Also, this choice depends upon the research objects that are available. These objects thus become at once explanans and explanandum, given the effort that goes into *constructing* those objects in the first place (Knorr Cetina 1999).

The contextual character of inquisitiveness becomes even clearer if we think of what it takes to develop oneself as an inquisitive researcher: not only should the institutional atmosphere in some way be conducive to that, it also requires that one is trained into recognizing the interesting scientific challenges. What is more, curiosity can only persist if there is a legitimacy to trying out possible dead ends and failures. It has in fact been demonstrated that current levels of competition and the pressure of acquiring scarce resources lead researchers to avoiding such risks (Moore et al. 2017). Thus, the realization of the value of inquisitiveness is dependent on infrastructures such as funding and research agendas that enable it, but also on how the local practice allows and even values failure. The extent to which a researcher is free to be inquisitive, depends on the hierarchical position one is placed in, and how such hierarchies work in a specific practice. And what is valued as an interesting research problem is similarly inscribed not in the rules, but rather in the unwritten value schemes that circulate in the practice. To see inquisitiveness merely as aspirational would be to disregard this complexity. And to explain this contextual complexity, it is not enough to only look at the institutional arrangements.

#### Reflexivity

Consoli (2006) argues that scientists should have *reflexivity*, or the capacity to think about their own work from an external perspective, in view of the broader context to which their work connects. This reflexivity is needed to be able to deal with the moral complexities that research work inevitably comes with. To a large extent, along the lines of Consoli’s discussion, the moral thinking that reflexivity requires can be delivered by an individual person. Nonetheless, it is also self-evident that moral thinking can be supported by training as well as peer-discussion, and both depend on what is done and not done in the direct research environment, and how such critique is valued. Are the customs of the practice such that there is space—in terms of time and place, but also psychological safety—to conduct such reflection? Are the meanings that circulate in the practice sufficiently open-ended to make engagement sensible, or are they rather fixed and hostile to reflection? These are clearly questions of collectiveness, practice and culture, not (merely) individual or institutional matters.

#### Collegiality and Trust

The need for a good collegial context and the duty to preserve that context is often mentioned. In fact, this is exactly one of the guises in which the unspecific notions of culture often appear from which this analysis started. This lack of specificity may contribute to the seemingly self-evident appearance as not-institutional and hence aspirational, but such a conclusion cannot be drawn before looking in more detail to the constituting values.

One thing that its slightly more specific is the value of *trust*. The German Research Foundation DFG (1998) emphasizes the need for trust in the relations scientists build within their community, where building trust consists of maintaining clear and transparent procedures, accuracy in attribution and citation, and accessibility of securing facilities such as counselling and report. It also posits trust to be a necessary condition for any self-regulation of science to emerge. Yet, in contrast, Stroebe et al. (2012) have argued that such self-regulation, chiefly based on principles of peer-review and replication, are insufficient to prevent fraud, and have indeed failed so in notorious cases.

Remarkably, both understandings are elaborated as more or less “manageable” issues, i.e., through procedures. Alternatively, in view of our discussion above of the concepts of practices and culture, trust could be seen as a relation between persons and groups of persons, that consists of the belief that the other party in that relation is truthful and well-meaning. The extent to which such belief can emerge, depends on how people behave in daily practice, the narratives they repeat about what they think is important, and the responsibilities they avow to take. In some contexts, trust will primarily be conferred to one’s equals, and in other contexts more along hierarchical lines up and down. Or it may in some contexts more than others be connected to merit and the credit one person has acquired with the other.

One specific guise in which collegiality appears is in the duty of peer review. It is mentioned widely as a core aspect of preserving the quality of scientific knowledge (Spier 2006; Hangel and Schickore 2017). In order to contribute to the progress of science, peer review should be done in a critical but fair and constructive way. Ripley et al. (2012) argue that teaching peer review is generally recognized as an important element of mentorship. Interestingly, they also argue that such mentorship could do with further training support for the mentors. Several sources (Bohannon 2013; Ioannidis 2005) show that peer review in practice drops the ball quite often, and fails to single out all instances of bad science. It is also biased, notably against interdisciplinarity and against diversity and inclusion (Rafols et al. 2012; Moore et al. 2017, p. 3).

A problem such as the bias against interdisciplinary research can only be understood as defects of the research culture: along the lines discussed, it reproduces itself independently of both the positions of single researchers and institutional rules. Trying to resolve this through further rules and regulations seems futile, and also it seems not a matter of individual peer reviewers having bad intentions. Rather, it requires active reflections on how things are done and valued, and how responsibility is distributed.

#### From Aspiration to Culture

We started from the working hypothesis that aspirational values are more open to interpretation and more difficult to manage than sanctionable values, and therefore more likely to end up as individual responsibilities. However, in the exemplary values discussed here, it becomes clear that this attribution of responsibility is again complex, and by no means maps onto the individual-institution dichotomy. For their substantiation, the aspirational values are dependent on the practice and how people act, know and value within it. At the same time, it appears that this dependency is less clear than with the sanctionable values, and the elements related to culture and practice are less tangible.

## Building Responsible Cultures and Practices

Our analysis started with an articulation of some elements that relate to RI in culture and practice. We subsequently explored how values that are central to RI can be thought to map onto such a field of culture and practice halfway the scale between individuals and institutions. What does this imply for *achieving* RI? What interventions are opened up by these insights? Where should they be developed, and by whom? What does it mean to *build cultures and practices of research integrity?* The current analysis makes clear that the specific times and places that are connected to practices, and the specific content of cultures, are important objects on which this integrity work is to operate, rather than the abstract notions of culture and practice themselves.

Gunsalus (1993) already articulated that achieving RI is not only about having the appropriate regulations in place, but also about the leadership of an institution “walking the talk” (see also Mejlgaard et al. 2020 for a recent reflection on this) and expressing the value of acting ethically. However, this idea of “walking the talk” solves a different problem than does introducing the idea of *integrity work* as presented by Breit and Forsberg (2016): the former is about identifying leadership examples as specific normative sources, the latter is about recognizing the dynamics of different normative sources and different types of normative source, and the fact that they are never “finished” and permanently in need of attention. Or put in the terms developed in this article, some of the ethics of RI needs to be about “caring for the research practice” (cf. Davies 2019, who reports that researchers do in fact recognize and articulate this need). It involves taking into account how certain social and cultural processes may become institutionalised and thus normalised and taken for granted (cf. Powell and DiMaggio 1991).

First, the elements of practice are themselves direct points of interventions. Skills are important in the makeup of practices, and we have seen that much of RI depends on them (see above: peer review, methodology and its presentation, etc.). Also, mentorship has been identified as a vital mechanism of transferring skills, but it needs attention how what is transferred in mentorship circulates further through the practice. And even though “technological fixes” for moral problems such as automated plagiarism checks have historically proven naïve (Johnston 2018; Sarewitz and Nelson 2008), there might nonetheless be realistic pointers to technical or procedural interventions.

Second, there is the issue of where the interventions are to be made. Given that human action is influenced by more complex sources than (individual) ethics and (institutional) rules and regulations, the question of “how to achieve better integrity” will hardly be answered by “more ethics” or “more rules and regulations”. Being articulate about practice and culture in this differentiated way might refine future interventions. One important site of intervention that does emerge from the vocabulary of practices and culture, and which has so far received little attention in the RI discourse, is the realm of *meaning*. While it is far from straightforward how meaning can be an object of intervention, we also cannot do without it. The questions of what authorship means and what trustability means are vital. While asking these questions could be part of the *reflexivity* that Consoli (2006) calls for, our analysis adds that this reflexivity should not only concern individual conduct and motivations, but also the practice at large, including the elements of which it is built: skills and routines by which people do things, technologies and other contextual arrangements that allow people to do some things and not others, value systems that are in place in both formal and informal ways, and the written and unwritten hierarchies with ensuing distributions of accountability and responsibility. That is: how things are done and known, how they are valued, and how people account for their actions and the conduct of scientific research.

This requires both a philosophical sensitivity among research practitioners and a sociological sensitivity on the part of research administrators and mentors. They need to be able to articulate and convey what is to be done, what is good research, as well as supply this knowledge with an account of how this is both a matter of individual duties and of collectively maintaining the practice as such. This goes beyond institutional provision of training. Krstic (2015) argues that such efforts should be both aimed at, and arranged by, early career academics. This group is at once most vulnerably positioned in power relations, and in the epistemic position to identify those vulnerabilities. The vocabulary of cultures and practices developed here is seems then appropriate to articulate those relations.

The notion of *integrity work* (Breit and Forsberg 2016) must in our view be situated at exactly this level of culture and practices. Breit and Forsberg use the word “work” to literally refer to activities undertaken either by individuals or by institutions to get integrity in place: e.g. making ethical decisions, organizing integrity courses, and offering resistance against pressures towards compromising of integrity. In light of the current analysis, this idea merits further expansion: it is not only the actions that matter, but also what kind of world is both reflected and constructed through these actions. Culture and practice do need a similar approach to be part of the integrity work that they are referring to. The current analysis thus also responds to the call by Clegg et al. (2007) for a further development of the notion of “ethics as practice” to reflect the contextual and dynamic nature of research integrity, and the fact that rule-based ethics typically fails to capture the intricacies of making choices on the work floor.

## Conclusion and Reflection

Starting from the observation that in discussions on RI, notions of “culture” and “practice” are underdeveloped, the current discussion provides a further conceptualization of these notions. Connecting them to values that typically circulate in RI discussions, these conceptualizations of practice and culture were carried towards possible implementations of *integrity work*.

Implementation of culture starts with awareness of the role of culture. Interestingly, a quantitative survey recently found that culture plays a considerable role in the occurrence of questionable research practices (Haven et al. 2020) and this potentially gives more reason to start working on other interventions that may foster a responsible research culture. This should entail RI-training, training of supervisors and mentors of PhD students, foster reflexivity at research departments; interventions that specifically address research culture. The current analysis provides further advice on the direction in which such development can be sought.

One question to reflect upon at this point is whether culture is a meaningful and necessary addition to thinking through research integrity. Indeed, Gläser et al. (2015) argue that culture is superfluous as an explanatory factor. However, in the current argument, the term is not used as an explanation (*explanans*), but rather as the thing that needs to be explained (*explanandum*) and something that can serve as a site of intervention. Combined with the mapping that was provided to values that are central to RI, the notions of practice and culture have been elevated well above triviality. We believe that this is a valuable contribution to the RI repertoire and should be part of discussions about RI.

It is likely that people will keep using the notions of culture and practice in an unreflexive and sweeping way, i.e. using it exactly as an explanans and not as an explanandum. The concepts are vulnerable to such usage, because they can easily be captured: no one will contest the general statement that a “good practice” and a “culture of safety” are desirable (even if such usage only serves very particular interests, as observed in medical-ethical debates on "human dignity", see Macklin 2003; Pinker 2008). This is one additional reason why formal training in research integrity needs to address what we mean when we use philosophical and practice-theoretical terms such as culture and practice. In concrete cases of such sweeping usage, it seems there are two possible remedies. If the usage is in fact correct, it may require specification in the terms developed above. If it is incorrect and the usage in fact obfuscates ambiguous or unspecific policy, it should be dropped in favour of concrete measures and clear and unambiguous attributions of responsibility.

In conclusion, cultural and practice theory can enrich the discourse on RI. Policy as well as training should pay attention to measures that can help foster a responsible research culture, which includes paying attention to the important values that constitute culture, to how these values influence practices of researchers, and to how they can be targeted for interventions. It helps to look at how values relate to the way people know things in practice, how they value them, how they do things, and how they attribute responsibility. In any case, it is high time to abandon vague references to culture. Our framework provides the initial tools to do so. However, future research should further explore how the notions of culture and practice can take a more prominent role in the debate on Research Integrity.

## Footnotes

1 RI is often equated with *Responsible Conduct of Research* (RCR), even though the former is strictly a somewhat more moral and abstract concept, while the latter would refer to a more positive and concrete specification of what “is and is not done”. Also, both RI and RCR are often just assumed to amount to the absence and active elimination of *Questionable Research Practices* (QRPs) (Tijdink et al. 2014; Fanelli 2009; Steneck 2006), *Sloppy Science* (Harris 2017), and fraud understood as *Falsification, Fabrication and Plagiarism* (FFP) (ALLEA 2017, p. 8; Breit and Forsberg 2016, p. 3; Al-Amad 2017). Also, Research Ethics has been suggested as differing from Research Integrity by its focus on building a good reputation for science, while the latter focuses on the commitments and responsibilities of the individual (Spier 2006, p. 190). While all these notions highlight slightly different problems and coping strategies, we reckon them part of the same overarching discourse of RI, which we will thus use as the main term throughout. Unless stated otherwise, our use of RI comprehensively refers to all these issues.

2 The boundary between the two categories is in reality not that clear cut. see also Shaw (2019), who shows that the notion of individual responsibility is already problematic at the philosophical level.
